# Genome Sequence of the Multiple-β-Lactam-Antibiotic-Resistant Bacterium *Acidovorax* sp. Strain MR-S7

**DOI:** 10.1128/genomeA.00412-13

**Published:** 2013-06-27

**Authors:** Takamasa Miura, Hiroyuki Kusada, Yoichi Kamagata, Satoshi Hanada, Nobutada Kimura

**Affiliations:** Bioproduction Research Institute, National Institute of Advanced Industrial Science and Technology (AIST), Tsukuba, Ibaraki, Japana; Graduate School of Life and Environmental Sciences, University of Tsukuba, Tsukuba, Ibaraki, Japanb; Bioproduction Research Institute, National Institute of Advanced Industrial Science and Technology (AIST), Toyohira, Sapporo, Hokkaido, Japanc

## Abstract

*Acidovorax* sp. strain MR-S7 was isolated from activated sludge in a treatment system for wastewater containing β-lactam antibiotic pollutants. Strain MR-S7 demonstrates multidrug resistance for various types of β-lactam antibiotics at high levels of MIC. The draft genome sequence clarified that strain MR-S7 harbors unique β-lactamase genes.

## GENOME ANNOUNCEMENT

Antibiotics are some of the most widely prescribed drugs in modern medicine. Improper use or prolonged use of antibiotics such as in medical treatment and stock farming has led to strains of bacteria that develop resistance to antibiotics (1, 2). In general, four types of antibiotic resistance mechanism are known, i.e., production of antibiotic-degrading or -modifying enzymes (3–5), mutation of active sites (6, 7), presence of antibiotic efflux pumps (8, 9), and formation of biofilm to prevent antibiotics from penetrating (10).

*Acidovorax* sp. strain MR-S7 was isolated from activated sludge from the treatment of wastewater containing β-lactam antibiotics. According to 16S rRNA gene sequence analysis, we placed strain MR-S7 in the class *Betaproteobacteria*, with 97.4% similarity to the validly described species *A. temperans* strain PHL (11), and located a different clade among the 10 strains with deposited genomic data, *Acidovorax* sp. KKS102 (NC_018708), CF316 (NZ_AKJX01000236), NO1 (EU521706), and JS42 (CP000539); *A. citrulli* AAC00-1 (CP000512); *A. avenae* subsp. *avenae* ATCC 19860 (AF078759) and RS-1 (JN601517); *A. radicis* N35 (HM027578); *A. ebreus* TPSY (CP001392); and *A. delafieldii* 2AN (HM625980).

Strain MR-S7 showed high resistance to a variety of β-lactam antibiotics, for example, MICs for ampicillin, amoxicillin, cephalexin, and cefadroxil in strain MR-S7 were ≥500 mg/liter. In this study, the draft genome sequencing of strain MR-S7 was performed, and the genes involved in multiple-β-lactam-antibiotic-resistant activity were identified. The whole genome of strain MR-S7 was sequenced by paired-end sequencing on an Illumina Hiseq2000 sequencing system provided by the Hokkaido System Science Co., Ltd. (Sapporo, Hokkaido, Japan). This sequencing run yielded 21,966,696 high-quality filtered reads with 101-bp paired-end sequencing, providing approximately 440-fold genome coverage. Using the Velvet program with a hash length of 75 bp (12), these reads were assembled into 105 contigs and 12 scaffolds (split into 130 contigs), of which the average length was 42,811 bp. Prediction of protein-coding sequences (CDS) and annotation were performed by the Microbial Genome Annotation Pipeline (http://www.migap.org/), which utilizes MetaGeneAnnotator (13), RNAmmer (14), tRNAScan-SE (15), and BLAST (16). The draft genome sequence of strain MR-S7 comprised 5,007,754 bp, with a GC content of 68.3%. The genome contained 4,772 putative CDSs, 51 tRNAs, and 3 rRNAs.

Eighteen putative β-lactamase domain-containing proteins, 12 multidrug efflux pump proteins, and 8 penicillin-binding proteins were found in strain MR-S7. A protein BLAST (BLASTp) search using the NCBI database showed that the average value of the sequence identity of putative β-lactamase proteins was 70%, while the average values of the sequence identities of the multidrug efflux pump proteins and penicillin-binding proteins were 87%. The relatively low sequence identities of putative β-lactamase proteins suggested that the diverse resistance mechanisms in strain MR-S7 have not been acquired by lateral gene transfer in the recent past, but that the acquisition occurred in ancient times, and that the genes responsible for antibiotic resistance have independently evolved into strain MR-S7.

### Nucleotide sequences accession numbers.

The draft genome sequence of *Acidovorax* sp. strain MR-S7 has been deposited in the DDBJ/EMBL/GenBank database under accession number BANP00000000. The version described in this paper is the first version, BANP01000000.

## References

[1] Mak JK Kim MJ Pham J Tapsall J White PA 2009 Antibiotic resistance determinants in nosocomial strains of multidrug-resistant *Acinetobacter baumannii*. J. Antimicrob. Chemother. 63:47–54 18988680 10.1093/jac/dkn454

[2] Froggatt JW Johnston JL Galetto DW Archer GL 1989 Antimicrobial resistance in nosocomial isolates of *Staphylococcus haemolyticus*. Antimicrob. Agents Chemother. 33:460–466 2729941 10.1128/aac.33.4.460 PMC172460

[3] Wright GD 2005 Bacterial resistance to antibiotics: enzymatic degradation and modification. Adv. Drug Deliv. Rev. 57:1451–1470 15950313 10.1016/j.addr.2005.04.002

[4] Babic M Hujer AM Bonomo RA 2006 What’s new in antibiotic resistance? Focus on beta-lactamases. Drug Resist. Update 9:142–156 10.1016/j.drup.2006.05.005 16899402

[5] Riesenfeld CS Goodman RM Handelsman J 2004 Uncultured soil bacteria are a reservoir of new antibiotic resistance genes. Environ. Microbiol. 6:981–989 15305923 10.1111/j.1462-2920.2004.00664.x

[6] Lowy FD 2003 Antimicrobial resistance: the example of *Staphylococcus aureus*. J. Clin. Invest. 111:1265–1274 12727914 10.1172/JCI18535 PMC154455

[7] Ubukata K Nonoguchi R Matsuhashi M Konno M 1989 Expression and inducibility in *Staphylococcus aureus* of the mecA gene, which encodes a methicillin-resistant S. aureus-specific penicillin-binding protein. J. Bacteriol. 171:2882–2885 2708325 10.1128/jb.171.5.2882-2885.1989 PMC209980

[8] Yamane K Wachino J Suzuki S Kimura K Shibata N Kato H Shibayama K Konda T Arakawa Y 2007 New plasmid-mediated fluoroquinolone efflux pump, QepA, found in an *Escherichia coli* clinical isolate. Antimicrob. Agents Chemother. 51:3354–3360 17548499 10.1128/AAC.00339-07 PMC2043241

[9] Okusu H Ma D Nikaido H 1996 AcrAB efflux pump plays a major role in the antibiotic resistance phenotype of *Escherichia coli* multiple-antibiotic-resistance (Mar) mutants. J. Bacteriol. 178:306–308 8550435 10.1128/jb.178.1.306-308.1996 PMC177656

[10] Drenkard E Ausubel FM 2002 Pseudomonas biofilm formation and antibiotic resistance are linked to phenotypic variation. Nature 416:740–743 11961556 10.1038/416740a

[11] Wen A Fegan M Hayward C Chakraborty S Sly LI 1999 Phylogenetic relationships among members of the *Comamonadaceae*, and description of *Delftia acidovorans* (den Dooren de Jong 1926 and Tamaoka et al. 1987) gen. nov., comb nov. Int. J. Syst. Bacteriol. 49 (Pt 2):567–576 10319477 10.1099/00207713-49-2-567

[12] Zerbino DR Birney E 2008 Velvet: algorithms for de novo short read assembly using de Bruijn graphs. Genome Res. 18:821–829 18349386 10.1101/gr.074492.107 PMC2336801

[13] Noguchi H Taniguchi T Itoh T 2008 MetaGeneAnnotator: detecting species-specific patterns of ribosomal binding site for precise gene prediction in anonymous prokaryotic and phage genomes. DNA Res. 15:387–396 18940874 10.1093/dnares/dsn027 PMC2608843

[14] Lagesen K Hallin P Rødland EA Staerfeldt HH Rognes T Ussery DW 2007 RNAmmer: consistent and rapid annotation of ribosomal RNA genes. Nucleic Acids Res. 35:3100–3108 17452365 10.1093/nar/gkm160 PMC1888812

[15] Lowe TM Eddy SR 1997 tRNAscan-SE: a program for improved detection of transfer RNA genes in genomic sequence. Nucleic Acids Res. 25:0955–0964 10.1093/nar/25.5.955 PMC146525 9023104

[16] Altschul SF Gish W Miller W Myers EW Lipman DJ 1990 Basic local alignment search tool. J. Mol. Biol. 215:403–410 2231712 10.1016/S0022-2836(05)80360-2

